# Structured Reactors Based on 3D Fe/SiC Catalysts: Understanding the Effects of Mixing

**DOI:** 10.1021/acs.iecr.2c01611

**Published:** 2022-08-08

**Authors:** Gonzalo Vega, Asuncion Quintanilla, Pablo López, Manuel Belmonte, Jose A. Casas

**Affiliations:** † Department of Chemical Engineering, Universidad Autónoma de Madrid, Campus de Cantoblanco, C/Francisco Tomás y Valiente 7, 28049 Madrid, Spain; ‡ Institute of Ceramics and Glass (ICV-CSIC), Campus de Cantoblanco, C/Kelsen 5, 28049 Madrid, Spain

## Abstract

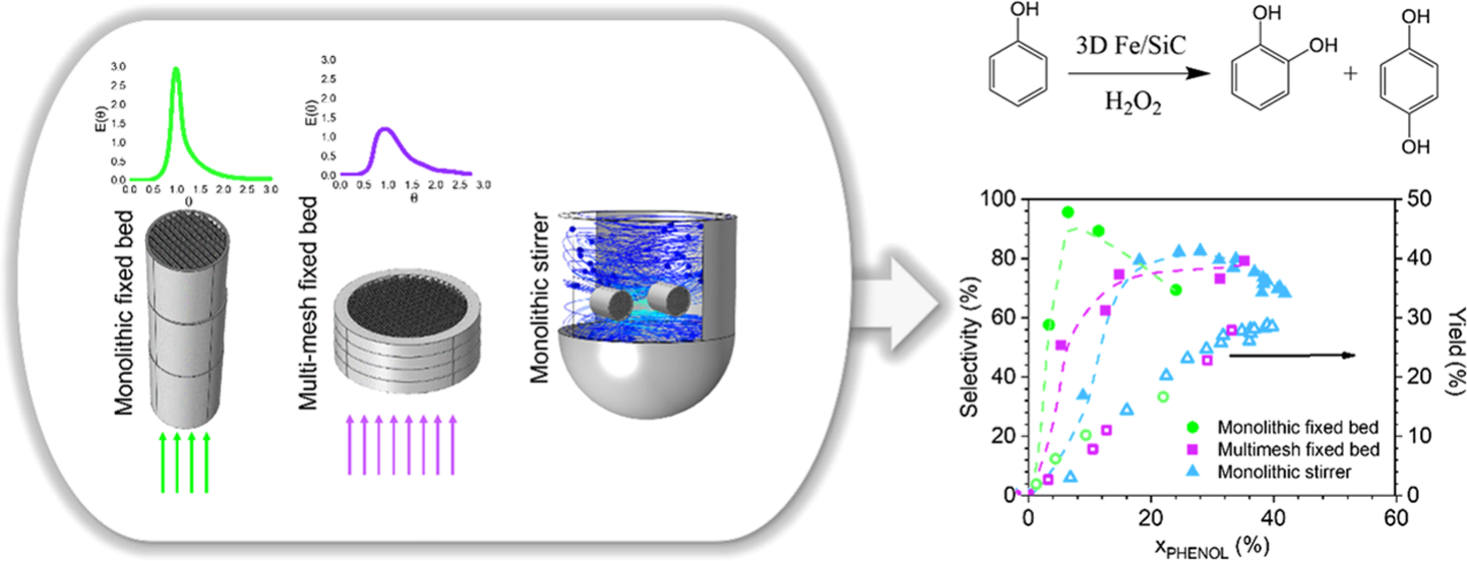

The application of structured reactors provides a number of advantages in chemical processes. In this paper, two different three-dimensional (3D) Fe/SiC catalysts with a square cell geometry have been manufactured by Robocasting: monoliths (*D* = 14 and *H* = 15 mm) and meshes (*D* = 24 and *H* = 2 mm) and studied in the catalytic phenol oxidation by hydrogen peroxide (H_2_O_2_) for the sustainable production of dihydroxybenzenes (DHBZ). The fluid dynamics, catalytic performance, reaction rates, external mass transport limitation, and catalyst stability have been compared in three different reactors, monolithic fixed-bed reactor, multimesh fixed-bed reactor, and monolithic stirrer reactor, at selected operating conditions. The results show that the mechanical stirring of the 3D Fe/SiC monoliths avoids the external mass transfer limitation caused by the presence of oxygen bubbles in the channels (produced from the HO*_x_*· species in autoscavenging radical reactions). In addition, the backmixing has a positive effect on the efficient consumption of H_2_O_2_ but an adverse effect on the phenol selectivity to DHBZ since they are overoxidized to tar products at longer contact times. On the other hand, the wall porosity, and not the backmixing, affects the susceptibility of the 3D Fe/SiC catalyst to the Fe leaching, as occurs in the mesh structures. In conclusion, the monoliths operating under plug-flow and external mass transfer limitation in the monolithic fixed-bed reactor (MFB) provide an outstanding phenol selectivity to DHBZ and catalyst stability.

## 1 Introduction

The structuring of the catalysts and reactors improves the chemical processes by increasing reaction rates, selectivities, and process efficiency.^1−3^ The structuring at the catalyst level leads to catalysts with appropriate diffusive lengths to reduce mass and heat transport limitations. However, the structuring at the reactor level leads to the use of a regular catalyst structure with low pressure drops, larger surface areas for solid–fluid contact, and thinner boundary layers that enhance the mass and heat transfer and precise control of the flow dynamics.^2^ Therefore, structured catalysts and reactors are one of the successful tools for process intensification to contribute to the sustainable development of the chemical industry.^4,5^ Numerous works in the literature illustrate the benefits that structured catalysts and reactors offer to process intensification.^3,4,6^ Their use implies the efficient utilization of materials and energy,^7,8^ as well as the reduction of toxic emissions and byproduct formation.^9,10^

The most common structured reactors are those equipped with well-designed structured catalysts, such as honeycomb, corrugated sheet, gauze, foam, fiber, or wire mesh packing. In a different level of application, microchannel reactors are also structured reactors where the catalytic materials are usually impregnated on a metallic plate installed inside the channel.^11^ Their limited scaling-up ability, among other features, prevents them from being used in industrial applications so far and is outside the scope of this study.

Honeycomb monoliths (a block with multiple straight parallel channels) are the most well-known structured catalytic reactors and are widely used in automobile and environmental applications. Their main disadvantage is the low radial heat and mass transfer rate, due to the laminar flow inside the channels, especially for ceramic monolithic supports.^2^

The recent irruption of the three-dimensional (3D)-printing technology in the field of catalysts has opened the doorway to a new generation of monolithic catalysts with nonconventional architectures to promote the fluid mixing to enhance the radial heat and mass transfer inside the structure while maintaining the advantages of the monoliths (low pressure drop and short diffusion lengths). In this sense, periodic open-cellular structures (POCs), 3D materials with an ordered assembly of interconnected regular unit cells with well-defined geometry^7,12−14^ and 3D monoliths with interconnected channels allowing the flow between adjacent channels^15−17^ are promising structures. The most challenging aspect is the development of printable inks containing the catalytic material and the further post-treatment of the as-printed scaffolds to provide the structures with adequate porosity (or accessibility to the active sites) while keeping adequate robustness for their catalytic application. This is already a reality in the printing of ceramic-supported catalysts using the Robocasting technique^18−20^ and also in the printing of catalytic metal alloys by the Selective Laser Sintering technology.^21,22^

In our previous works,^16,23,24^ 3D-printed 0.5wt%Fe/SiC honeycomb monoliths with interconnected channels and different cell geometries (square, troncoconical, and triangular) were manufactured by Robocasting and used as catalytic reactors in the hydroxylation of phenol by H_2_O_2_ to produce DHBZ in the aqueous phase. Our experimental^23,24^ and numerical research on computational fluid dynamics^16^ demonstrated that the triangular cell monoliths, with a higher macrochannel tortuosity, induced an oscillating flow inside the channel along with a transverse flow between adjacent parallel channels, resulting in a better overall performance. Phenol selectivity to DHBZ (*S*_DHBZ_) between 97.2 and 99.1% at phenol conversions (*X*_PHENOL_) ranging from 14 to 25% was obtained at a reaction temperature of 80 °C. These values were superior to those reported for the EniChem commercial process using the TS-1 zeolite in a slurry reactor (*S*_DHBZ_ = 90–95%, *X*_PHENOL_ = 20–25% at *T* = 80–100 °C) and also to those reported for other structured reactors, such as a wall microreactor^25^ and a submerged membrane reactor.^26^ Therefore, the 3D Fe/SiC monolithic reactors are novel intensified reactors with a high potential for the catalytic oxidation of hydrocarbons with H_2_O_2_. An aspect to be improved in these monolithic reactors, nevertheless, is the low-efficient H_2_O_2_ consumption. More than 70% of the H_2_O_2_ is consumed in nonproductive reactions such as the innocuous production of O_2_ and H_2_O (generated by the scavenging of the hydroxyl radical species, HO*_x_*·, coming from the decomposition of H_2_O_2_) and, to a lesser extent, in the production of tar byproducts coming from the overoxidation of DHBZ.^23^

In this line, the present work aims to find the most appropriate structured reactor for the catalytic hydroxylation of phenol to produce DHBZ. This reactor, while maintaining a good and stable overall performance, should enable a better efficient consumption of H_2_O_2_. For the latter, it is hypothesized that short contact times between H_2_O_2_ and the Fe catalyst will be preferable. For this reason, the following two reactors are proposed: (i) a monolithic stirrer reactor (MSR) to rotate the Fe/SiC monoliths already submerged in the reaction media at the appropriate stirring speed to achieve very high liquid velocities inside the channels,^27^ and (ii) a multimesh fixed-bed reactor (MMR), by the design and manufacture of novel Fe/SiC ceramic mesh catalysts (short-length monoliths) by the Robocasting technique. The fluid dynamics, catalytic performance, reaction rates, mass transport limitation, and also catalyst durability of both reactors have been compared to the conventional monolithic fixed-bed reactor (MFB).

## 2 Materials and Methods

### 2.1 3D-Printed Fe/SiC Catalysts

**Figure 1:**
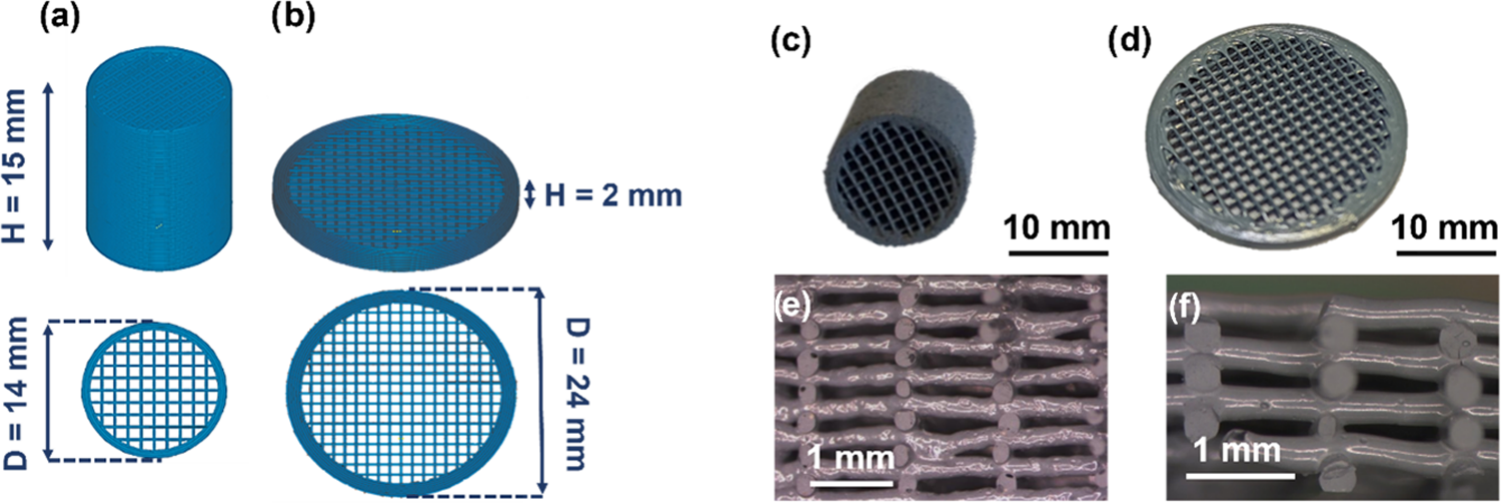
Computer design patterns (a, b), 3D cylindrical Fe/SiC structures after the thermal treatment (c, d), and cross-sectional optical views (e, f) for the 3D-printed honeycomb monolith (a, c, e) and mesh (b, d, f).

Two different 0.5wt%Fe/SiC cylindrical honeycomb structures, such as honeycomb monolith and honeycomb mesh, with square cell geometry and interconnected channels were 3D-printed using a three-axis robocasting system (A-3200, 3-D Inks LLC) at room temperature. The details of the Fe/SiC ink formulation are described elsewhere.^23^Figure 1a,b shows the CAD patterned structures designed and their external dimensions. The monolith and mesh differ in the height-to-diameter ratio (H/D), with the former being 10 times higher.

To achieve these geometries and dimensions, the monolith was formed by 60 layers with a linear array of parallel filaments in the *x*–*y* plane; each array rotated 90° with respect to the adjacent layers to produce an orthogonal pattern design, with a distance between in-plane adjacent rods (center-to-center) -*a*- of ∼1.3 mm, and an external wall formed by two outer rings for each layer. The mesh presented a larger diameter (24.3 *vs* 13.4 mm in the monolith), containing eight layers with an orthogonal pattern, in this case, *a* ∼1.2 mm, and had an external wall of five outer rings to enhance the mechanical resistance of this 3D architecture.

The as-printed scaffolds were subjected to different heat treatments, first to remove the organics used in the ink formulation (*viz* high and low molecular polyethylenimine and hydroxypropyl methylcellulose) by calcination at 600 °C in air for 2 h, and then, to provide the architectures with the adequate mechanical robustness, in this case, using a pressureless spark plasma sintering furnace (SPS-510CE Fuji Electronic Industrial Co., Ltd.) at 1200 °C in an argon atmosphere for 5 min.

The consolidated 3D-printed structures after the thermal treatment are shown in Figure 1c–f. Table 1 summarizes their main physical characteristics. The data provided are average values obtained from measuring five 3D structures. As expected, the pieces shrank during the thermal treatment. The mesh structure was more affected by the temperature than the monoliths. The wall thickness of the meshes is thinner than that of the monoliths, although the same tip inner diameter (330 μm) was used. Thus, the hydraulic diameter (*d*_H_) is slightly higher in the mesh (0.90 *vs* 0.88 mm), as well as the wall porosity (ε_wall_) (21 *vs* 16%) and the bulk density (1.3 *vs* 1.0 g cm^–3^). The external ring in the mesh has less contribution to the final structure, and consequently, the mesh structures have a lower channel interface area (*a*_v_) and a low specific surface area (*S*_BET_) than the monoliths. In any case, both structures retain their integrity while they are cut crosswise to show the faced lateral interconnections of the vertical channels (Figure 1e,f).

**Table 1:** Physical Properties of Robocast 3D Catalysts after Thermal Treatment^a^

geometry	monolith	mesh
*D* (mm)	13.4	24.3
*H* (mm)	14.8	2.04
*W* (g)	1.3	0.6
ρ_GEO_ (g cm^–3^)	0.6	0.6
ρ_BULK_ (g cm^–3^)^b^	1.05	1.28
ε_TOTAL_ (%)	59	66
ε_wall_ (%)	16	21
η (cell cm^–2^)	49	53
δ_WALL_ (μm)	298	258
*d*_H_ (mm)^c^	0.88	0.90
*a*_v_ (mm^–1^)	26.4	21.4
*S*_BET_ (m^2^ g^–1^)	39	25

a Dimensional parameters (diameter, *D*; height, *H*; and weight, *W*), geometrical and bulk densities (ρ_geo_, ρ_bulk_), open total and rod porosities (ε_total_, ε_wall_), cell density (η), wall channel thickness (δ_wall_), hydraulic diameter (*d*_H_), channel interface area (*a*_v_), and specific surface area (*S*_BET_) of the pieces used in reactions.

b ρ_bulk_ indicates the wall volume, equivalent to the particle density in pellets.

c Calculated as 4·*a*·P^–1^ (where *a* is the open channel area and *P* is the wetted perimeter).

The Fe content remains invariable and equal to 0.52 ± 0.03 wt % in both structured catalysts (measured by total reflection X-ray fluorescence, S2 PICOFOX TXRF spectrometer, Bruker Nano). Regarding the active catalytic phase, iron silicides, *viz*, Fe_3_Si and α-FeSi_2_, identified by Mössbauer spectroscopy, are considered as the iron catalytic species.^23^

Different techniques were employed as tools to characterize the fresh and used 3D catalysts in powdered form (obtained by crushing the scaffolds). The specific surface area (*S*_BET_) and external area (*A*_ext_) were determined using the nitrogen adsorption/desorption isotherms recorded at −196 °C employing a Micromeritics Tristar 3000 apparatus. Before the measurement, the samples were outgassed at 120 °C overnight. The elemental composition was measured in a LECO CHNS-932 analyzer. In addition, differential thermal analysis and thermogravimetry (DTA/TGA) were performed in a TA Instruments Discovery STD 650 under an air atmosphere with an air flow of 90 mL min ^–1^ at a heating rate of 10 °C min^–1^ from 30 to 950 °C.

### 2.2 Catalytic Reactors for Phenol Hydroxylation

The DHBZ production by phenol hydroxylation with H_2_O_2_ in an aqueous phase over 3D Fe/SiC catalysts has been studied at the laboratory scale in three different isothermal reactors: a monolithic fixed-bed reactor (MFB), a multimesh fixed-bed reactor (MMR), and a monolithic stirrer reactor (MSR). The MFB and MMR are flow reactors operated in continuous mode, while the MSR is operated batchwise. For the former reactors, the structured catalytic bed (consisting of several stacked 3D Fe/SiC catalytic structures) was settled on a small bed of spherical quartz beads (three layers of 1 mm diameter spheres) placed in a double-jacketed glass tube (GE Healthcare) with an internal diameter of 16 and 26 mm, respectively, according to the diameter of the monoliths and meshes (Table 1). For the MSR (a double-jacketed glass reactor with 1 L of capacity), the monoliths are mounted on the stirrer shaft as impeller blades. The stirrer shaft was constructed in glass to hold one monolith block on each side. The monoliths are placed at 65 mm from the top, 50 mm from the bottom, and 19 mm from the internal wall of the vessel. Photographs of the reactors are provided in Figure S1 of the Supporting Information.

For the MFB and the MMR, the feed liquid stream, containing the selected amount of phenol and H_2_O_2_ dissolved in water, was preheated and passed in up-flow through the fixed-bed at a constant flow rate using a piston pump (Shimadzu LC-20AD). The product stream was cooled down in a (homemade) steel heat exchanger, collected, and analyzed. The reaction was conducted until the steady-state was reached, requiring from 2 to 6 h of operation depending on the space time (τ) used. The hydroxylation reaction was performed under the following operating conditions: *C*_PHENOL,0_ = *C*_H2O2,0_ = 0.33 M, *T* = 80 °C, τ = *W*_CAT_*Q*_L_^–1^ = 0–254 g_CAT_ h L^–1^. Long-term experiments (72 h time on stream) were also carried out under the same operating conditions but at 254 g_CAT_ h L^–1^ for the MFB and 77 g_CAT_ h L^–1^ for the MMR. Water was always used as a heating fluid to maintain the desired reaction temperature (also in the MSR reactor).

The MSR was filled with 700 mL of 0.33 M phenol solution and stirred under 250 rpm. While the desired reaction temperature was reached, 25 mL of H_2_O_2_ (30 wt %) was added with a syringe. The samples were taken at different reaction times until the total consumption of H_2_O_2_ was reached. To enable the comparison, operating conditions as similar as possible to those used in the fixed-bed reactors were selected: *C*_PHENOL,0_ = *C*_H2O2,0_ = 0.33 M, *T* = 80 °C, although obviously the catalyst concentration inside the reactor (*C*_CAT_) was far lower in the MSR (4 *vs* ∼1000 g_CAT_ L^–1^). Catalyst stability was studied by reusing the monoliths in subsequent cycles.

To study the influence of the backmixing, an additional reactor was configured by the separation of the meshes with inert fixed beds made of quartz beads (two layers of 1 mm diameter spheres between meshes). This new reactor is denoted as a separated multimesh fixed-bed reactor (S-MMR). In addition, to study the mass transfer effect in the reaction performed under stirring, in the MSR, a control experiment was carried out using the catalyst in powder form, obtained by crushing the 3D Fe/SiC monoliths. In this case, the MSR was transformed into a slurry reactor (SR). The main characteristics of the five different reactors finally used are summarized in Table S1 of the Supporting Information.

On the other hand, the conversion (*X*) of reactants (denoted by i: phenol or H_2_O_2_) and the phenol selectivity (*S*), and yield (*Y*) to the identified products (j: CTL, HQ, RSL, or BQ) were calculated as follows
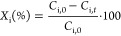
1
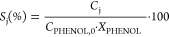
2
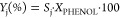
3where *C* is expressed on a mole basis and subscripts 0 and *t* stand for initial and a given reaction time, respectively.

The selectivity of the unidentified products, named as tar, was calculated as
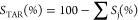
4

In addition, the efficiency of the H_2_O_2_ consumption was calculated from the hydroxylation products as
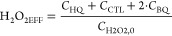
5

### 2.3 Residence Time Distribution

The backmixing degree in the fixed-bed reactors was studied by the experimental measurement of the residence time distribution (RTD). The reactors were loaded with inert 3D structures made from a polylactic acid (PLA) filament (PRUSA i3 MK3S+ printer) with the same geometry and dimensions as the 3D Fe/SiC catalysts to mimic the fluid dynamic conditions of the hydroxylation reaction. Bromophenol blue (BB) was used as an inert tracer. The RTD curve for the MFB was already obtained in previous work.^24^ Herein, the RTD curve of the mesh reactors, *viz*, MMR and S-MMR, has been measured by analyzing the response to a step tracer experiment. The reactor was filled with water. At *t* = 0, a bromophenol blue solution (*C*_BB,0_ = 0.0075 g L^–1^) was fed to the reactor at *Q*_L_ = 0.5 mL min^–1^ and ambient temperature. The samples were collected at the reactor exit until the concentration of the dye was similar to the concentration at the entrance.

From the resulting temporal absorbance profiles of the dye tracer at the reactor exit, the outlet *E*(*t*) curves were calculated as^28^
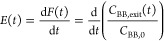
6

From the *E*(*t*) curves, the mean residence time, *t*_m_, and the variance, σ_t_^2^, were calculated as the first and second moment of the *E*(*t*) curve, respectively
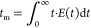
7
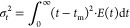
8

The Péclet number (*Pe*) is calculated by the following expression, only valid when *t*_m_ differs from the ideal residence time (calculated as *V*_L_*Q*_L_^–1^), which indicates the presence of dead or stagnant zones in the reactor^28^
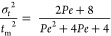
9

At low *Pe* numbers (<100), the axial dispersion is mainly controlled by the convection in the channel, and then the presence of a radial concentration profile is expected in laminar flow reactors. Meanwhile, at high *Pe* numbers (>100), the axial dispersion is mainly controlled by molecular diffusion, and the reactor can be considered as a plug-flow reactor.

To compare directly the flow performance inside reactors of different sizes, a normalized RTD curve was used, named as *E*(θ)
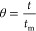
10
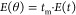
11

### 2.4 Analytical Methods

The progress of the reaction was followed by periodically taking liquid samples from the reactors. Phenol and the aromatic byproducts, *viz*, HQ, CTL, BQ, and RSL, were determined by high-performance liquid chromatography (Ultimate 3000, Thermo Scientific, C18 5 μm column 150 × 4.6 mm^2^, 4 mM H_2_SO_4_ as the mobile phase, and a DAD detector at wavelengths of 210, 246, and 246 nm). The H_2_O_2_ concentration was determined using the TiOSO_4_ method in a Cary 60 UV–vis spectrophotometer at a wavelength of 410 nm. Furthermore, the BB tracer concentration was also measured using a Cary 60 UV–vis spectrophotometer at a wavelength of 591 nm. Finally, the content of Fe in solution was measured by the atomic absorbance spectroscopy method (Analytic Jena NovAA 400P).

### 2.5 External Mass Transfer Analysis

The Carberry number (*Ca*) was calculated in each reactor at the different reaction conditions employed. The criteria used for negligible external mass transfer limitation under steady-state condition states that the *Ca* number must be smaller than 0.05.^29^

The *Ca* number is defined as the ratio between the observed reaction rate for reactants, (−*r*_i_) in mol L^–1^ s^–1^ and the maximum external mass transfer rate
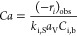
12where *a*_v_ is the channel interface area (in m^–1^, see Table 1), *C*_i,b_ is the concentration (in mol L^–1^) of the reactants (i, such as phenol and H_2_O_2_) in the liquid phase, and *k*_i,S_ is their corresponding L–S mass transfer coefficient (m s^–1^). Analogous to eq 12, the *Ca* for CTL and HQ products was calculated by considering the observed reaction rates for products (*r*_j_)_obs_ and their corresponding concentrations in the liquid phase (*C*_j,b_). *k*_i,S_ and *k*_j,S_ were estimated by the empirical correlations proposed for microreactors (*Re* < 200)^29^
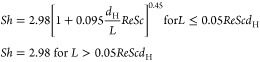
13where 2.98 is the shape factor for square channel geometries, and *L* is the reactor length.

### 2.6 Reaction Rates

The mass balance of the reactant (i) in the fixed-bed reactor, assuming isothermal plug-flow and absence of reaction in the liquid phase, can be expressed as
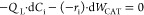
14where (−*r*_i_) is the reaction rate of i reactant in mol g_cat_^–1^ h^–1^. Considering that *Q*_L_ remains constant, and the definition of τ, eq 14 can be expressed as
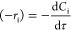
15

Analogously, the reaction rate of j products (*r*_j_), also expressed in mol g_cat_^–1^ h^–1^, is
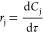
16

On the other hand, the mass balance of the reactant (i) in a batch stirrer reactor, in the absence of reaction in the liquid phase, can be expressed as
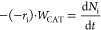
17where *N*_i_ is the number of moles of i and *t* is the residence time in min. Considering that the liquid volume remains constant and the definition of *C*_CAT_, eq 17 can be expressed as
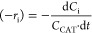
18

Analogously, the reaction rate of j products (*r*_j_), also expressed in mol g_cat_^–1^ h^–1^, is
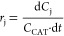
19

The reaction rate expressions were initially assumed to be the same as those reported for the 3D Fe/SiC monolithic catalysts,^24^ since the same catalyst is employed with different physical structures and in a different reactor
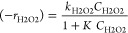
20
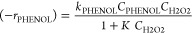
21
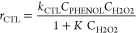
22
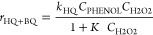
23

As can be seen, the H_2_O_2_ decomposition occurs according to a Langmuir–Hinshelwood–Hougen–Watson kinetic model (eq 20), while the phenol hydroxylation (eq 21), as well as CTL (eq 22) and HQ (eq 23) production, to an Eley–Rideal kinetic model.

In this work, the data analysis was carried out using the OriginLab 2017 with the initial conditions: *C*_PHENOL_ = *C*_PHENOL,0_, *C*_H2O2_ = *C*_H2O2,0_, and *C*_CTL,0_ = *C*_HQ,0_ = *C*_BQ,0_ = 0 at *T* = 80 °C. To solve the differential equations, the classical fourth-order Runge-Kutta method was used in conjunction with Levenberg–Marquardt algorithm for chi-square (χ^2^) minimization, which is obtained by dividing the residual sum of squares (RSS) by the degrees of freedom. The model discrimination was based on statistical analysis, considering the minimum RSS value and the coefficient of determination (*R*^2^) closer to 1, and also taking into account the physical meaning of the estimated parameters.

## 3 Results and Discussion

### 3.1 Structured Fixed-Bed Reactors

**Figure 2:**
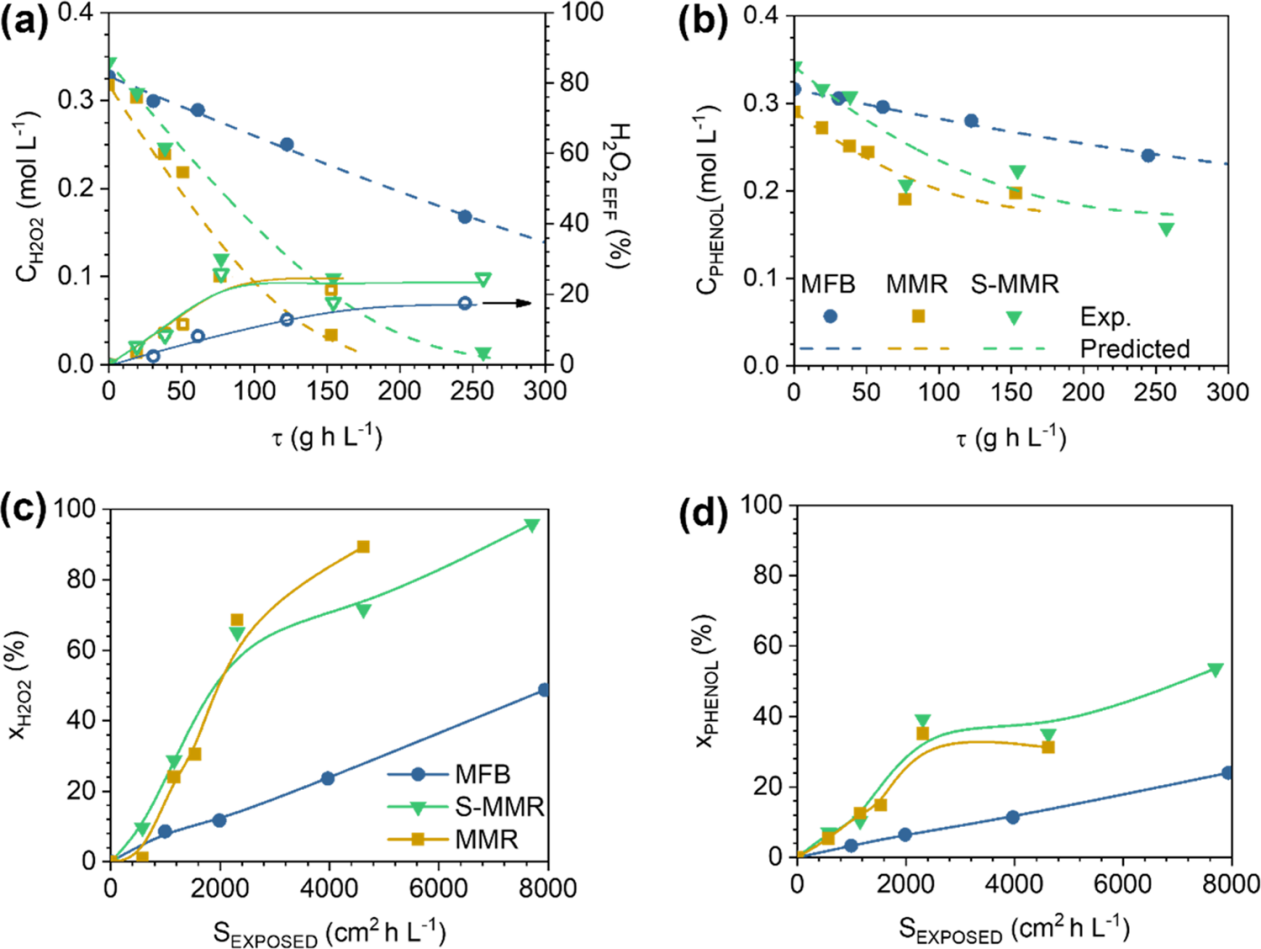
Evolution of H_2_O_2_ concentration and efficiency (a) and phenol concentration (b) with space time and evolution of H_2_O_2_ (c) and phenol (d) conversion with the exposed surface area using different 3D Fe/SiC catalytic reactors. Operating conditions: *C*_PHENOL,0_ = *C*_H2O2,0_ = 0.33 M, *T* = 80 °C, and τ = 0–254 g_CAT_ h L^–1^. Dashed lines in (a) and (b) are the predicted data by the kinetic equations of Table 3 for the MFB and the MMR.

**Figure 3:**
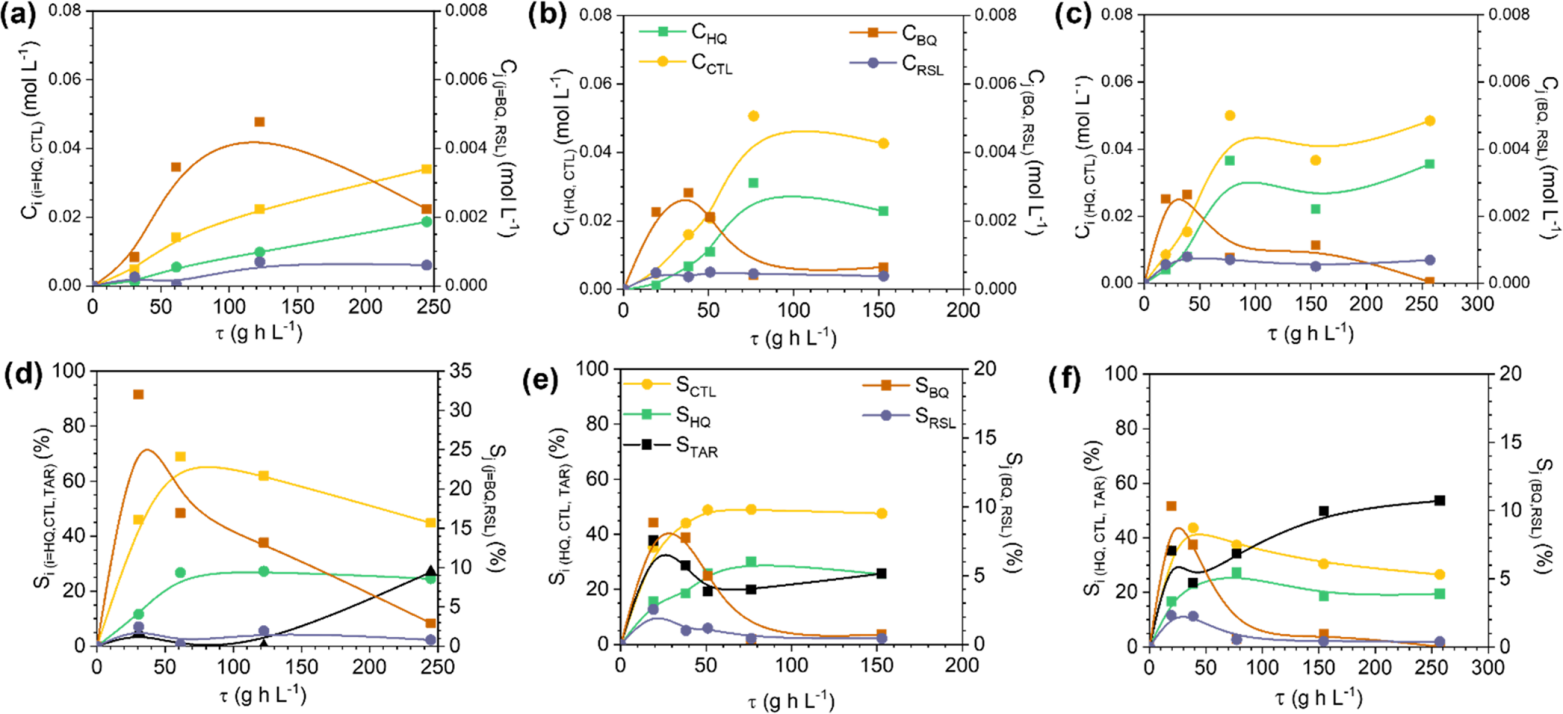
Evolution of product concentrations (a–c) and selectivities (d–f) with space time in the MFB (a, d), MMR (b, e), and S-MMR (c, f). Operating conditions: *C*_PHENOL,0_ = *C*_H2O2,0_ = 0.33 M, *T* = 80 °C, and τ = 0–254 g_CAT_ h L^–1^.

The results obtained in the phenol hydroxylation reactions using the monolithic (MFB) and the multimesh reactors (MMRs) at 80 °C are provided in Figures 2 and 3. As can be seen in Figure 2a, the H_2_O_2_ decomposition occurs faster in the MMR reactor and, consequently, also the phenol hydroxylation reaction (Figure 2b). Thus, at short space time values, such as τ = 147 g_CAT_ h L^–1^, almost complete H_2_O_2_ consumption (*X*_H_2_O_2__ > 94%) is achieved in the MMR, while only around 20% has been consumed in the MFB. Anyway, at the end of the reaction (when H_2_O_2_ is completely consumed), a similar phenol conversion is achieved in both reactors, around 24%. The representation of the reactant conversions *vs* the exposed surface in terms of cm^2^ h L^–1^ (calculated considering the *a*_v_ of each structure), which is pertinent considering the different surface areas of the 3D Fe/SiC catalyst in both reactors (Table 1), confirms that the performance of the MMR is superior to that of the MFB, although the same 0.5wt%Fe/SiC catalyst is used.

These results point out that there is an additional contribution to the heterogeneous reaction occurring on the catalyst surface in the MMR. In fact, Fe was detected in the MMR reactor effluent in an appreciable concentration, from 2 to 15 ppm, depending on the liquid flow used and the operation time of the meshes. In contrast, the Fe concentration detected in the MFB effluents was always lower than 1 ppm. Therefore, the improved performance of the MMR, particularly on the H_2_O_2_ decomposition reaction, may be assigned to the catalytic effect of the Fe leached in the liquid phase.^30^Figure S2 of the Supporting Information shows a linear relationship between H_2_O_2_ or phenol conversion and the concentration of leached Fe in the liquid phase for the MMR.

As it was already demonstrated in one of our previous studies,^19^ the Fe leaching in the 3D Fe/SiC catalyst can be attributed to the wall porosity of the structure rods. A high ε_wall_ not only implies better accessibility to the active sites but also enhances the susceptibility to Fe leaching. This is the case of the mesh structures compared to the monoliths.

Figure 3a,b shows the concentration profiles of the reaction products as a function of space time. In general, the concentration of the oxidized products is higher in the MMR than in the MFB, since in the former, the reaction takes place more rapidly due to the catalytic contribution of the Fe in solution. Thus, the consumption of the H_2_O_2_ results in greater efficiency (H_2_O_2EFF_) in the mesh reactor (see Figure 2a). This was the main aspect to improve with respect to the MFB, although, still, the H_2_O_2_ efficiency remains low, ∼25%.

Regarding the DHBZ production (Figure 3), the main species obtained in both reactors are CTL and HQ, while BQ and RSL are present in low concentrations. As expected, BQ behaves as an intermediate product, showing a maximum in the concentration profile at a lower space time in the MMR than in the MFB, since the reaction progresses faster in the former. The BQ rapidly gives rise to HQ, and it is less accumulated in the reaction media.^23^ Consequently, HQ is present in higher concentrations in the MMR, and then the CTL/HQ molar ratio is affected, observing an average value of 1.7 in the MMR *vs* 2.7 in the MFB. In spite of this, the phenol selectivity to HQ is similar in both reactors, *S*_HQ_ ∼ 25%, and, interestingly, the selectivity to catechol is significantly higher in the MFB than in the MMR, *S*_CTL_ = 75 *vs* 50% (Figure 3d and e). This decrease in selectivity in the MMR is due to the presence of tar species that remain even at a total consumption of H_2_O_2_ (Figure 3d). Therefore, the overoxidation of any aromatic species to produce tar occurs to a greater extent in the MMR than in the MFB (*S*_TAR_ = 25 *vs* 0% at 147 g_CAT_ h L^–1^). In principle, this tar is produced in the liquid phase.^23,31,32^ The liquid volume in the MMR reactor is lower than that in the MFB. Therefore, other aspects apart from the liquid volume play a role in the production of tar. For instance, the Fe in solution may promote this undesirable reaction or also the different flow patterns inside the reactors. The 10 times higher value of the H/D ratio in the MFB compared to the MMR may affect the selectivity.

**Figure 4:**
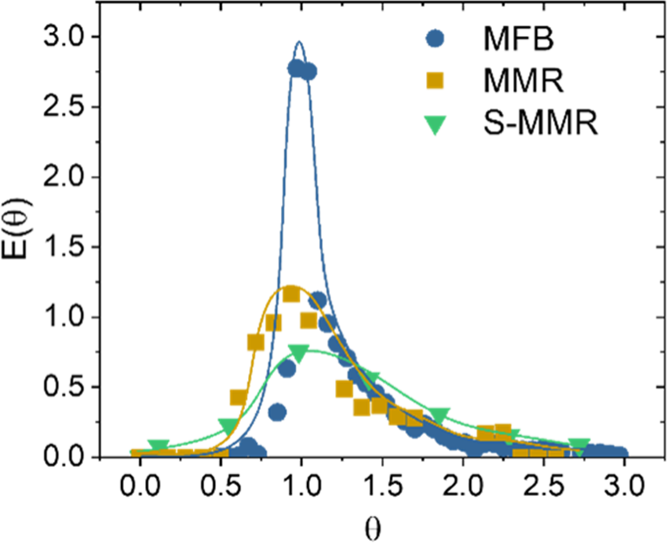
Experimental *E*(θ) curves at *Q*_L_ = 0.5 mL min^– 1^ in the different 3D Fe/SiC catalytic reactors.

Figure 4 shows the normalized RTD curve for the MFB and MMR at *Q*_L_ = 0.5 mL min^–1^. It evidences the higher dispersion in the MMR. The *Pe* numbers are 12 for the MMR and 198 for the MFB; the former is a backmixing-bed reactor (note that *Pe* = 0 is for ideal stirrer reactors), while the latter is a plug-flow reactor. This can be attributed to the high internal reactor diameter in the MMR (24 *vs* 15 mm in the MFB) that provokes backmixing and the presence of stagnant fluid (eq 7).

To gain an insight into the effect of backmixing on the reactor performance in the DHBZ production, an S-MMR has also been studied. The height and the bed porosity (or liquid volume) of the mesh reactor were increased by including a quartz beads-packed bed between two meshes; in this way, the H/D value was 0.8 (between 3.1 for the MFB and 0.3 for the MMR, Table S1). As can be seen in Figure 4, the flow mixing inside the S-MMR was even higher than that in the MMR (Figure 4), with the Pe number being 2. It seems that the presence of the fixed quartz beads contributes to the stagnation of the liquid.

The results obtained in the hydroxylation of phenol performed in the S-MMR have been included in Figures 2 and 3. The H_2_O_2_ and phenol concentrations decrease faster than those in the MFB but slower than those in the MMR (Figure 2a,b), in accordance with the concentration of leached Fe, from 1 to 7 ppm, with intermediate values between those obtained in the MFB and the MMR. A linear relationship between the H_2_O_2_ or phenol conversion and the leached Fe was also observed (Figure S2 of the Supporting Information). Thus, the lower *X*_H2O_2__ observed upon exposed surface than that in the MMR (Figure 3d) is due to the less contribution of the leached Fe to the H_2_O_2_ decomposition. This lower Fe in solution does not seem to affect the phenol hydroxylation reaction (Figure 2d). The products in the S-MMR are present in a concentration similar to that in the MMR (Figure 3). However, a higher selectivity to the tar product is now obtained, as high as 50%, in detriment of the *S*_CTL_ and *S*_HQ_, now being as high as 25 and 20%, respectively. Therefore, a higher backmixing and a higher liquid volume of the S-MMR (three times as much as the liquid volume than the MMR, Table S1) favor the tar production. The tar comes from the overoxidation of the aromatic species in the liquid phase, and the higher residence time caused by the mixing and the presence of stagnant zones contribute to the extension of this undesirable reaction.

Table 2 summarizes the Fe leaching, the *S*_TAR_ at τ = 147 g_CAT_ h L^–1^, and also the percentage of carbon adsorbed on the catalyst surface (%TOC_adsorbed_) at the range of τ = 0–254 g_CAT_ h L^–1^, along with the Pe in the three fixed-bed reactors studied. The carbon has been calculated as the difference between the carbon content in the feed and effluent, quantified using a TOC analyzer (Shimadzu TOC VSCH). As can be seen, the amount of carbon adsorbed varies in accordance with the susceptibility for the tar production and the backmixing degree in the reactor, S-MMR > MMR > MFB. The Fe leaching, nevertheless, does not depend on the flow pattern, and it does not seem to catalyze tar production.

**Table 2:** Comparison of the Percentage of Leached Fe (%Fe_leached_), Selectivity to Tar (*S*_TAR_), Carbon Adsorbed on the Catalyst Surface (%TOC_adsorbed_), and Péclet Number (Pe) in the Different 3D Fe/SiC Reactors

type of reactor	%Fe_leached_	*S*_TAR_	%TOC_adsorbed_	*Pe*
MFB	5	0	5	198
MMR	25	20	19	12
S-MMR	15	50	22	2
MSR	3	30	26	
SR	2	30	21	

To sum up, the production of DBHZ from phenol hydroxylation by H_2_O_2_ takes place slower and is more selective in a monolithic reactor than in a multimesh reactor. The low DHBZ production rate in the presence of Fe/SiC monoliths is due to the absence of the homogeneous reaction caused by the Fe leached, since the concentration of Fe in the liquid phase is very low, while the high selectivity can be assigned to the plug-flow regime that disfavors the tar production. The H_2_O_2_ efficiency, however, is slightly increased in the multimesh reactor because the Fe in solution contributes to the production of DHBZ.

#### 3.1.1 Long-Term Experiments

**Figure 5:**
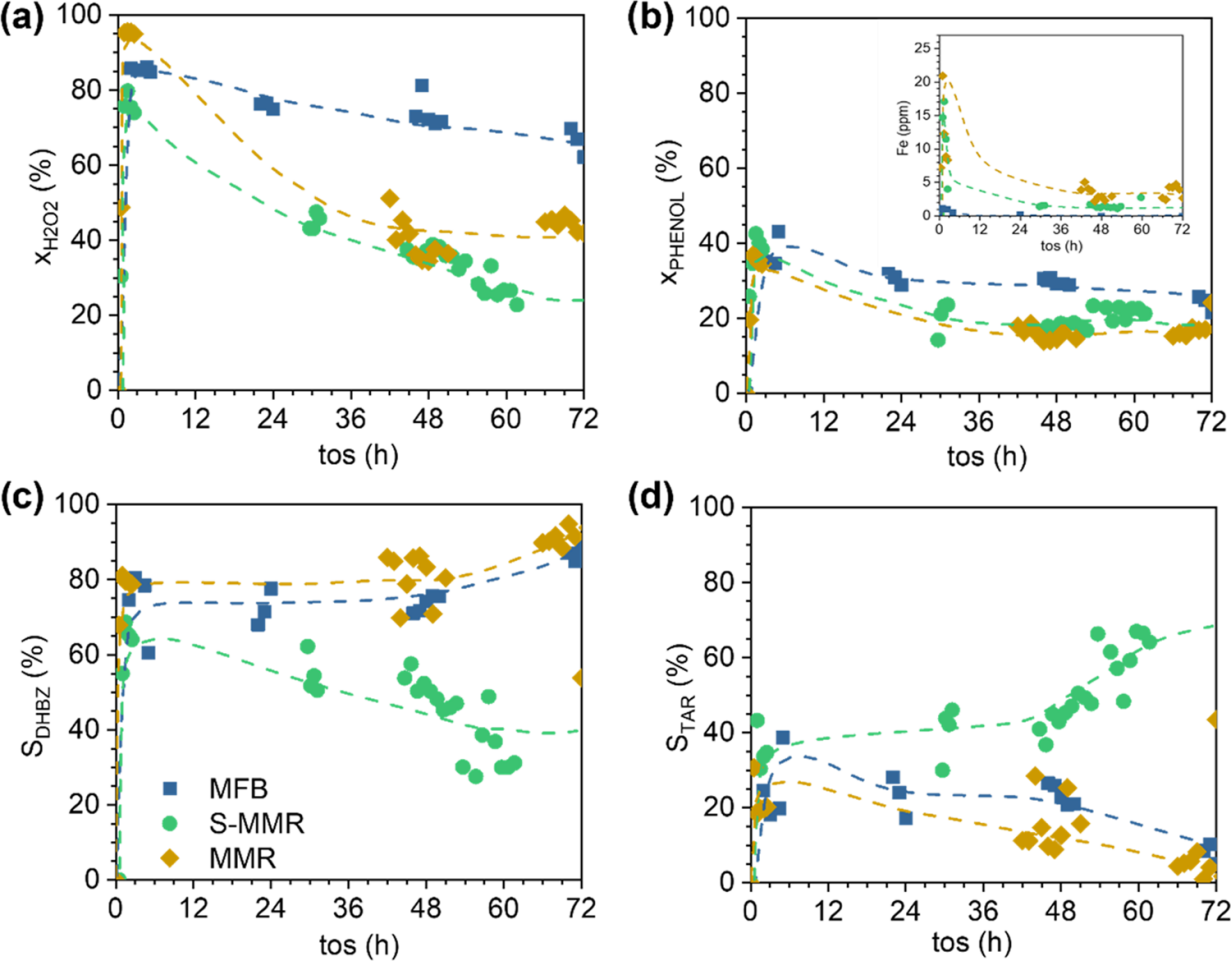
Profiles of H_2_O_2_ (a) and phenol (b) conversions and selectivity to DHBZ (c) and tar (d) in long-term experiments using different 3D Fe/SiC catalytic reactors. Operating conditions: *C*_PHENOL,0_ = *C*_H2O2,0_ = 0.33 M, *T* = 80 °C, and τ = 254 g_CAT_ h L^–1^ for the MFB and 77 g_CAT_ h L^–1^ for the MMR and the S-MMR.

The reactors loaded with 3D Fe/SiC structures were operated for 72 h at τ = 254 g_cat_ h L^–1^ for the MFB and 77 g_CAT_ h L^–1^ for the MMR and the S-MMR. The results in Figure 5 evidence that there is a fast and significant decrease in the H_2_O_2_ conversion (*X*_H2O2_) with the time on stream. As can be seen, *X*_H2O2_ decreases by 50% in the MMR and by 40% in the S-MMR during the first 36 h, while this decrease is gradual and slow (only by 10%) in the MFB after the 72 h on stream. This loss of activity correlates to the Fe leached to the reactor effluent (see the inset in Figure 5b), which is very significant at the initial reactor operation (up to 20 ppm of Fe), and it is stabilized after 36 h of stream when Fe concentrations lower than 5 ppm were measured. Since phenol conversion (*X*_PHENOL_) is less affected by the Fe leaching, its decay is similar in the three reactors, although the MFB maintains the highest conversion (Figure 5b). In addition, the *S*_DHBZ_ for the MFB and the MMR is stable at 80% during the long-term experiment, while for the S-MMR, the reactor that leads to the highest selectivity to tar products, the *S*_DHBZ_ decays by 45% (Figure 5c) accompanied by the significant appearance of tar in the reactor effluent (Figure 5d). The presence of deposits on the 3D Fe/SiC catalyst is also higher in the S-MMR. Table S2 of the Supporting Information summarizes the properties of the catalysts after the long-term experiments. The surface area does not significantly change, but a higher amount of carbon deposits can be found in the MMR and the S-MMR.

After testing for 72 h on stream, it is expected that the H_2_O_2_ and phenol conversions remain similar since the Fe leaching does not occur, but the *S*_DHBZ_, at some point, starts to continuously decrease due to the saturation of the 3D catalysts by these species and their consequent appearance in the liquid phase. In fact, this phenomenon was observed in the MFB after 8 days on stream in our previous work of Vega et al.,^23^ while in the S-MMR, it occurs after 48 h due to the higher production of tar in this last reactor.

### 3.2 Monolithic Stirrer Reactor

**Figure 6:**
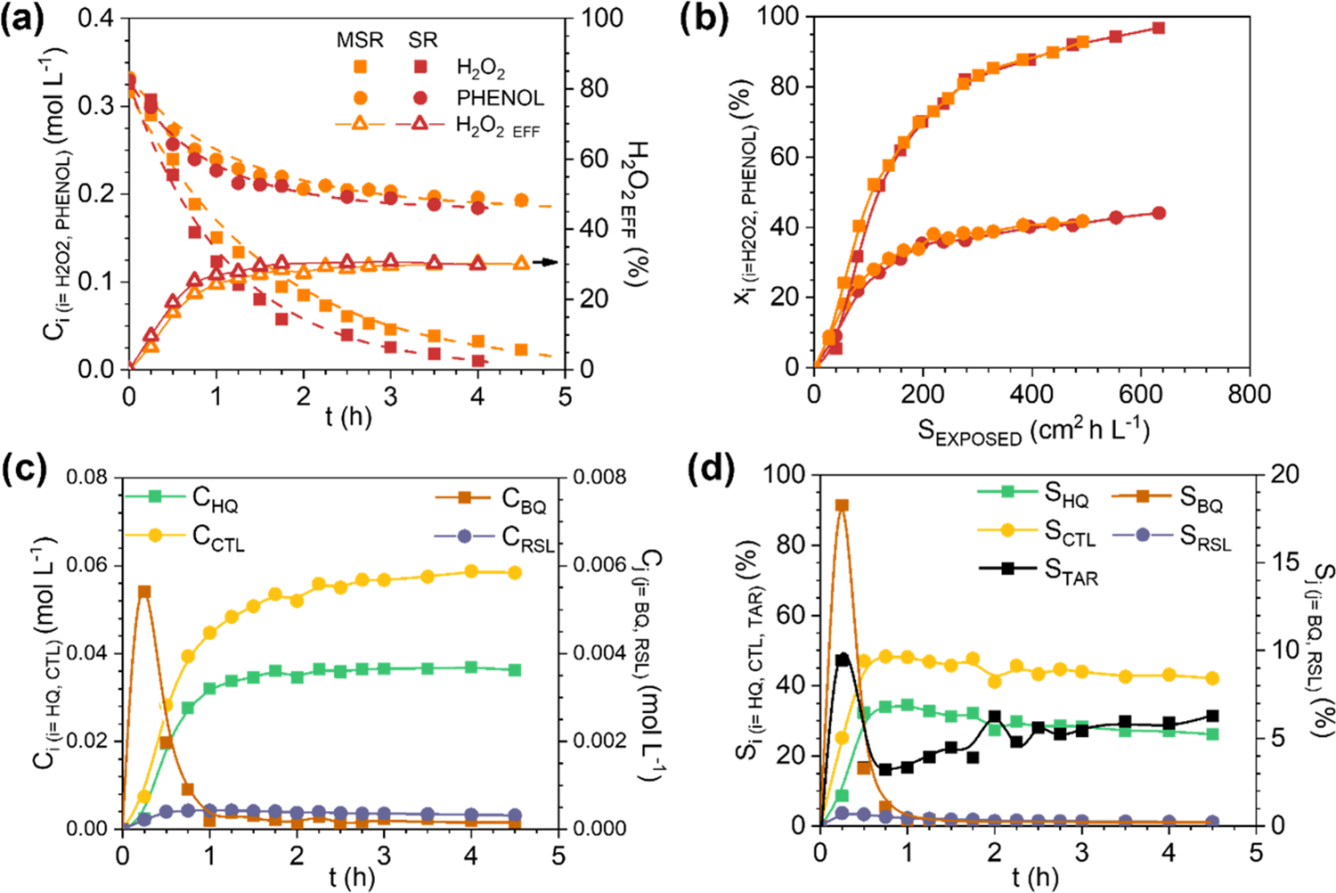
Temporal H_2_O_2_ and phenol concentrations and H_2_O_2_ efficiency profiles (a), phenol and H_2_O_2_ conversion profiles with the exposed surface area (b) in the monolithic stirrer reactor and slurry reactor, and product concentrations (c) and selectivity (d) with reaction time in the monolithic stirrer reactor. Operating conditions: *C*_PHENOL,0_ = *C*_H2O2,0_ = 0.33 M, *T* = 80 °C, and *C*_CAT_ = 3.55 g_CAT_ L^–1^. Dashed lines in (a) are the predicted data by the kinetic equations of Table 3 for the MSR.

A completely different catalytic reaction system is now tested. In the MSR, two pieces of 3D Fe/SiC monoliths were assembled as stirrer blades, submerged in 700 mL of 0.3 M phenol and H_2_O_2_ solution and rotated at 250 rpm. The results obtained in the hydroxylation of phenol are provided in Figure 6. Almost total consumption of H_2_O_2_ was achieved after 5 h of reaction, and the *X*_PHENOL_ reached was 42%, higher than that obtained in the MFB and the MMR (Figure 2). In addition, the CTL and HQ are produced in higher amounts than in the fixed-bed reactors, with the hydroxylation of phenol to HQ being more favored than that in the fixed-bed reactor, with a *S*_HQ_ of up to 35%. Therefore, the highest H_2_O_2_ efficiency is obtained under stirring (*viz*, H_2_O_2, EFF_ (%) = 20 for the MFB, 25 for the MMR, and 30 for the MSR) (see Figures 2a and 6a). In addition, the amount of leached Fe is less significant under stirring than in the fixed-bed reactors, particularly the mesh reactors (Table 2). Note that the catalyst concentration is 100 times lower in the MSR than that in the fixed-bed reactors (see Table S1). These results confirm that the reactant mixing benefits the efficient consumption of H_2_O_2_ because the hydroxylation reaction takes place faster, and it is not due to the Fe leached.

Regarding the tar species, they are present from the beginning of the reaction, and their concentration progressively increases to reach a *S*_TAR_ = 30% at total H_2_O_2_ consumption. This selectivity value is between that obtained in the MMR (*S*_TAR_ = 20%) and the S-MMR (*S*_TAR_ = 50%) in the first use. However, a larger amount of tar is present on the monolithic surface in the MSR than on the fixed-bed reactors (see %TOC_adsorbed_ in Table 2 and weight loss and amount of carbon in Table S2), which even causes a reduction in the specific and external surface area (Table S2). These results also support the fact that the tar production is not catalyzed by the presence of Fe in solution, which is far lower in the MSR than that in the fixed reactors (Table 2). The favored phenol polymerization reaction under a rich oxygen atmosphere in a stirrer reactor, with a high liquid-to-catalyst ratio compared to a fixed-bed reactor, has already been reported in the literature.^31,32^

**Figure 7:**
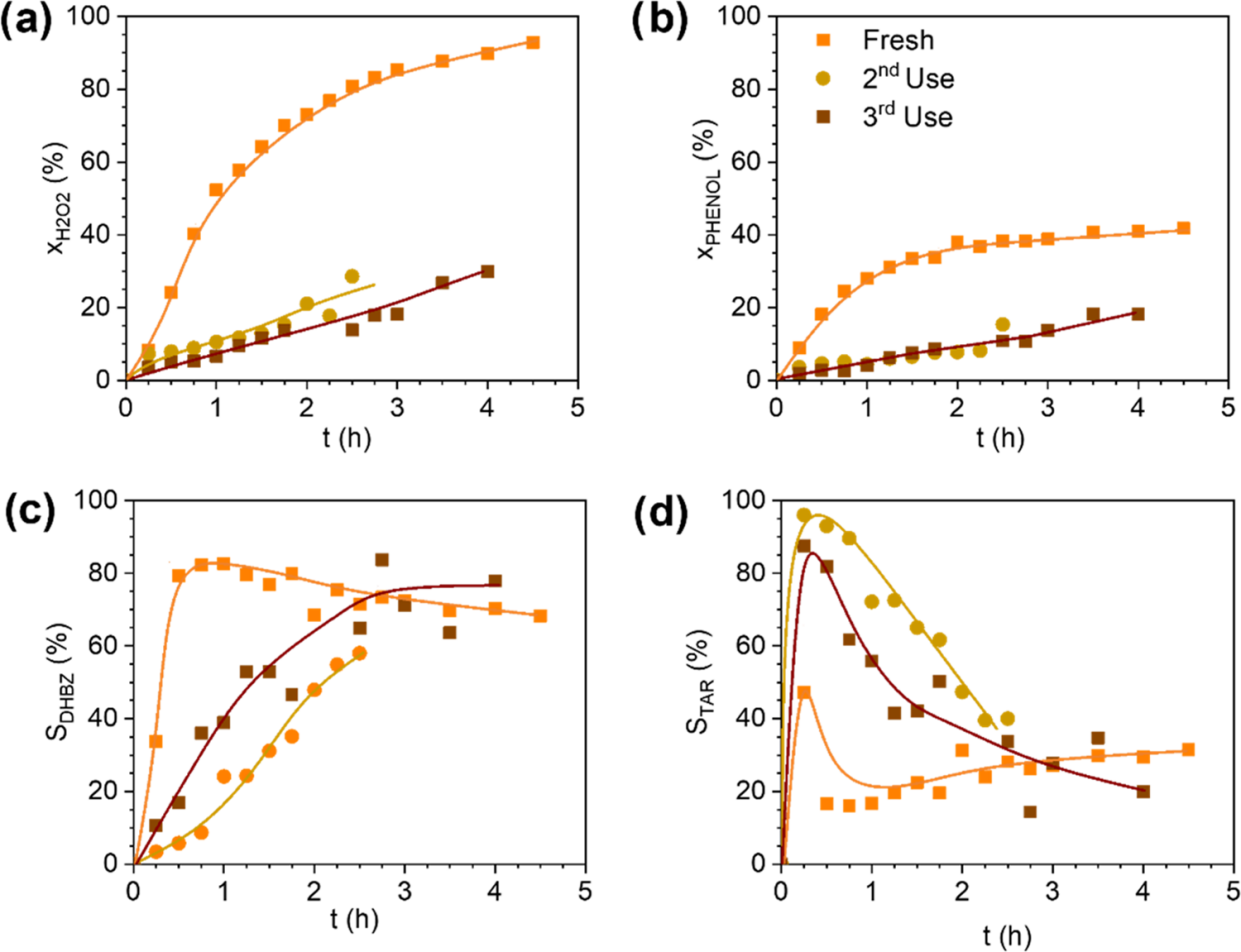
Profiles of H_2_O_2_ (a) and phenol (b) conversions and selectivity to DHBZ (c) and tar (d) in consecutive uses of the 3D Fe/SiC monoliths in the MSR. Operating conditions: *C*_PHENOL,0_ = *C*_H2O2,0_ = 0.33 M, *T* = 80 °C, and *C*_CAT_ = 3.55 g_CAT_ L^–1^.

The difference in the Fe leaching and production of tar in the MSR compared to the fixed-bed reactors will lead to a different durability of the 3D Fe/SiC catalyst in this reactor. Figure 7 shows the results obtained in consecutive uses of the monoliths in the MSR (pieces only washed with distilled water between cycles). As can be seen, the monolith activity dramatically decreases after the first use, exhibiting an 81 and 67% loss in the H_2_O_2_ and phenol conversion, respectively (Figure 7a,b). Likely, the carbon deposits on the catalyst surface after the first use (see Table S2) block the Fe active sites for the H_2_O_2_ decomposition into the oxidant radical species. Also, the tar species are present in a major amount in consecutive uses, especially at the beginning of the reaction. This points out that the tar species are washed out with the liquid reactants from the catalyst surface at the beginning of the reaction (Figure 7c,d).

### 3.3 Apparent Reaction Rates and Mass Transfer in the Different 3D-Structured Reactors

To compare the performance of the three reactors, *viz*, MFB, MMR, and MSR, the H_2_O_2_ consumption rate (−*r*_H2O2_), phenol oxidation rate (−*r*_PHENOL_), and catechol (*r*_CTL_) and hydroquinone (*r*_HQ_) production rates have been obtained. To this aim, the kinetic rate equations discriminated for the 3D Fe/SiC monoliths in the MFB, eqs 20–23, have been fitted to the experimental concentration profiles (Figures 2, 3, and 6). The resulting rates with the apparent kinetic rate constant values (*viz*, *k*_H2O2_, *k*_PHENOL_, *k*_CTL_, and *k*_HQ_) are summarized in Table 3 (the standard deviation values are provided in Table S3 of the Supporting Information). The kinetic parameter (*K*) that includes the H_2_O_2_ adsorption constant was maintained with the same value, *K* = 11.84 L mol^–1^, as the one estimated for monoliths in the MFB since the catalyst is the same in all of the reactors. Note that the apparent kinetic rate constant values are always higher for the MMR than for the MFB, as expected. These apparent values include the contribution of the homogeneous reaction catalyzed by the Fe leached. This contribution does not alter the overall reaction rates, and the good coincidence between the experimental (in symbols) and predicted (in lines) concentration profiles is illustrated in Figure 2, as well as by the parity plot shown in Figure S3 of the Supporting Information.

**Table 3:** Apparent Reaction Rates (in mol g_cat_^–1^ h^–1^) for Reactants and Products in the Phenol Hydroxylation with H_2_O_2_ over 3D Fe/SiC Catalysts in the Different Reactors^a^

reactor	(−*r*_H2O2_)	(−*r*_PHENOL_)	*r*_CTL_	*r*_HQ+BQ_
MFB				
MMR				
MSR	1.74·10^–1^*C*_H2O2_	3.36·10^–1^*C*_H2O2_*C*_PHEN_	1.48·10^–1^*C*_H2O2_*C*_PHEN_	1.10·10^–1^*C*_H2O2_*C*_PHEN_

a Concentrations in mol L^–1^.

In contrast, the kinetic rate equations discriminated for the 3D Fe/SiC monoliths in the MFB and the MMR do not fit the experimental concentration profiles obtained in the MSR. For the latter, the best-fit equations were a first-order kinetics for each reactant or product (see equations in Table 3, the predicted curves in Figure 6, and the parity plot of Figure S3). At first glance, these results would indicate that the chemical process in the fixed-bed reactors is limited by the external mass transfer. However, the *Ca* number is always below 0.05. The initial reaction rates are always lower than the external mass transfer rates for reactants and products (detailed calculations are provided in Tables S4 and S5 of the Supporting Information). This inconsistency indicates that the correlation employed for the calculation of the L–S mass transfer coefficient in microreactors, eq 13, is not applicable to our phenol hydroxylation reaction system: first, due to the unconventional geometry of the 3D-printed monoliths with interconnected channels, and second, because oxygen was produced upon reaction, as it was visualized inside the reactor exit tube, and therefore, a biphasic fluid with an unknown pattern is inside the channels. The oxygen is produced by the spurious consumption of H_2_O_2_ because of the scavenger reactions of the HO*_x_*· species, as has also been observed in previous works dealing with the catalytic phenol hydroxylation process.^33−35^

It is expected that the presence of the oxygen bubbles in the fixed-bed reactor channels hinders the access of H_2_O_2_ to the Fe active sites, and thus, they interfere with the mass transfer of the reactants and products. However, when the channels are rotated at a high stirring speed, as is the case in the MSR, the oxygen bubbles are not expected to interfere in the reaction since they rapidly leave the monoliths (the residence time of the liquid in the channels of the MSR is lower than 1 s as was calculated by computational fluid dynamics in similar monoliths,^27^ while in the MBF it is between 3 and 14 min). To confirm this, the same amount of catalyst is used in powder form (by crushing the monoliths pieces) and suspended in the liquid media as a slurry reactor (SR). The results provided in Figure 6a,b show that H_2_O_2_ and phenol conversion profiles similar to those of the MSR are obtained, particularly at the same exposed surface area of the catalyst used (Figure 6b). This demonstrates that the hydroxylation reaction proceeds in the absence of mass transfer limitation in the MSR, and thus the oxygen in the channels does not affect the reaction performance. Also, the amount of Fe leached was similar in both stirring reactors (Table 2).

**Figure 8:**
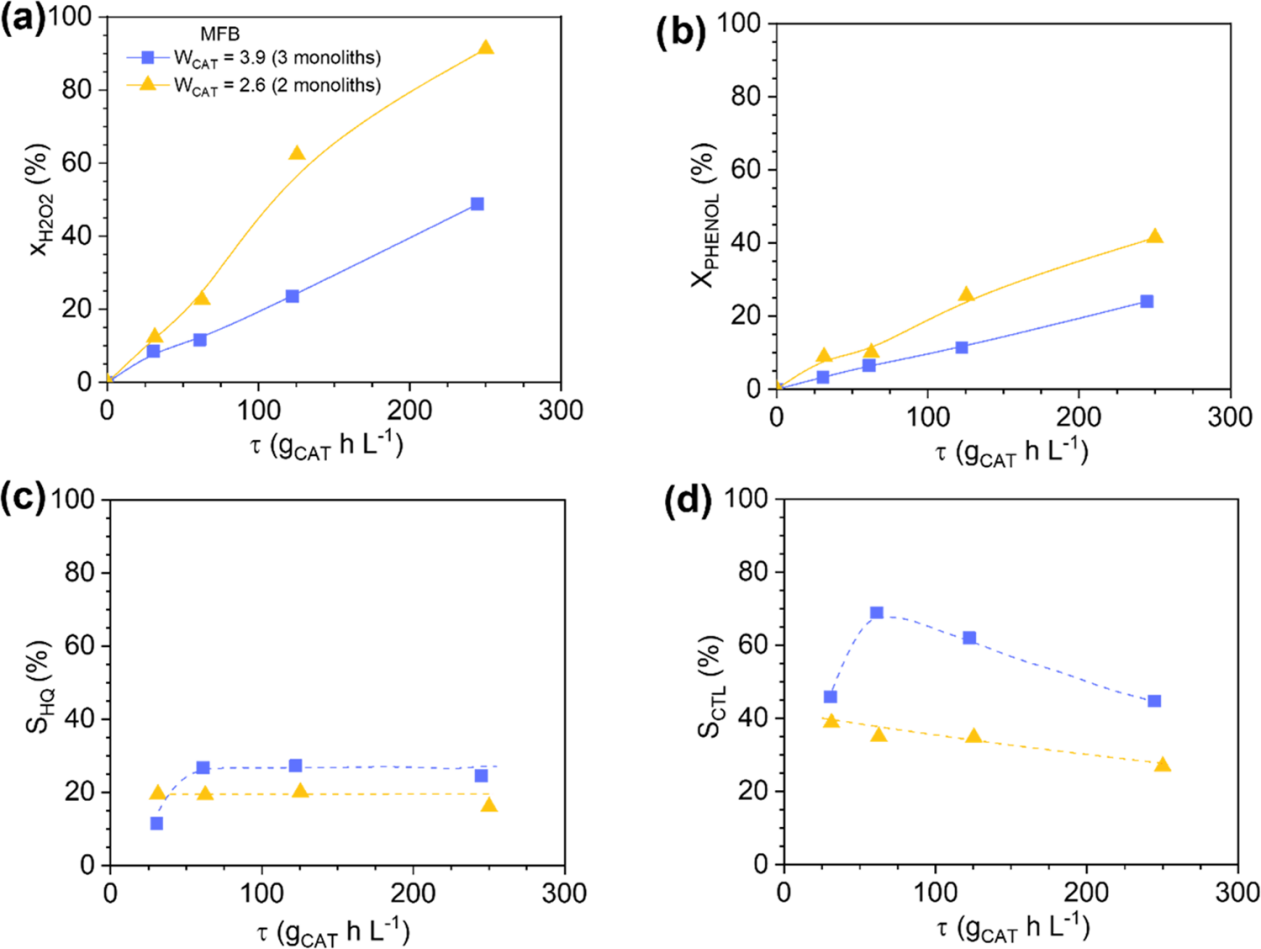
Effect of the liquid flow rate on the catalytic performance of the MFB: H_2_O_2_ (a) and phenol (b) conversions and selectivity to catechol (c) and hydroquinone (d). Operating conditions: Operating conditions: *C*_PHENOL,0_ = *C*_H2O2,0_ = 0.33 M, *T* = 80 °C, and τ = 0–254 g_CAT_ h L^–1^.

To gain an insight into the effect of the oxygen in the channels in the fixed-bed reactors, a second MFB loaded with two monoliths (instead of three pieces) was used and operated at the required flow rates to work in the space time selected for the hydroxylation of phenol. The results show some effect of the flow rate on the catalytic performance (see Figure 8). At a given space time and a lower flow rate (or catalytic bed consisting of two monoliths), higher H_2_O_2_ and phenol conversions and lower selectivity to CTL and HQ are achieved. If the external mass transfer limits the chemical process, the use of lower flow rates, at the same space times, would have an adverse effect on the reactant conversions. Therefore, the flow rate affects the hydroxylation performance but not according to the “conventional” mass transfer phenomena. A plausible explanation could be that the oxygen bubbles produced upon reaction leave the channels earlier when using two instead of three monoliths, and the H_2_O_2_ is then more accessible to the Fe active sites. This may be possible because the pressure drop is expected to be lower in the former due to the low flow rate and the amount of catalyst employed.^16^ On the other hand, the fraction of the liquid volume in the channels is expected to be higher when using two monoliths, and then, the tar production is favored in detrimental of the CTL and HQ selectivity. In conclusion, in the fixed-bed reactors, particularly in the MFB where the Fe leaching does not contribute to the chemical reaction rates, the external mass transport of species can be affected by the presence of the oxygen bubbles, which hinders the accessibility of the reactants (particularly, H_2_O_2_) to the active sites, and this leads to a chemical process in which the resistance of the H_2_O_2_ transport to the active sites is included in the denominator, as the kinetic equations reflect (see Table 3).

**Figure 9:**
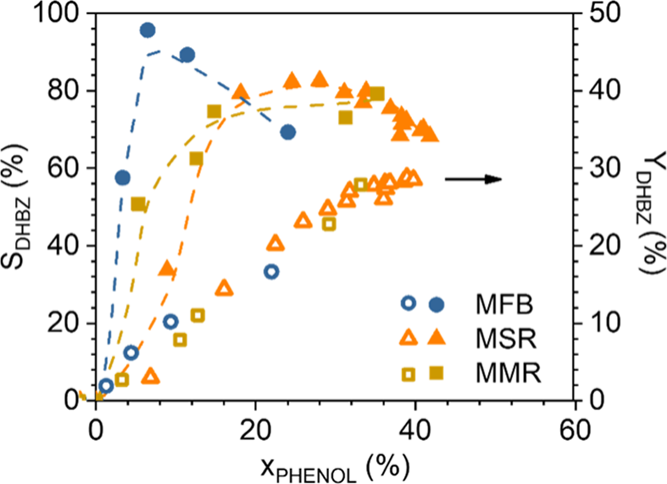
Comparison of the evolution of phenol selectivity and yield at *C*_PHENOL,0_ = *C*_H2O2,0_ = 0.33 M, and *T* = 80 °C.

Finally, Figure 9 compares the evolution of *S*_DHBZ_ and *Y*_DHBZ_ in the three reactors, *viz*, MFB, MMR, and MSR. The MFB provides an outstanding selectivity (*S*_DHBZ_ = 90–99%) at low *X*_PHENOL_ (from 6 to 10%, Figure 7b). However, the selectivity at high *X*_PHENOL_ (above 22%) remains higher in the MSR (*S*_DHBZ_ = 80%). In addition, the yield of desired DHBZ products in the MSR and the MMR can achieve superior values than in the MFB at the operating conditions selected in this study because the reaction progresses faster in the former due to the absence of mass transfer limitation (as occurring in the MSR) or the contribution of the Fe leached to the liquid phase (as in the MMR). Thus, for these two reactors, the lower selectivity can be compensated by the higher conversions achieved. A *Y*_DHBZ_ as high as 30% is the asymptotic value reached. This value is superior to those provided by the TS-1 catalyst in the commercial EniChem process (*S*_DHBZ_ = 24% at *T* = 80–100 °C). However, considering the 3D Fe/SiC catalyst stability, performing the reaction in the MFB assures a more prolonged reactor operation, while the use of the MSR implies more frequent catalyst recovery by washing the carbon deposits with a basic aqueous solution.^23^ The *Y*_DHBZ_ decreases up to 14% in the third use of the catalyst.

## 4 Conclusions

The performance of the hydroxylation of phenol with H_2_O_2_ over 3D Fe/SiC catalysts using different types of structured reactors with different flow patterns, such as the MFB (with a plug-flow), MMR (a backmixing flow), and MSR (perfect mixed flow), has allowed us to understand the effect of the mixing on the phenol hydroxylation reaction by H_2_O_2_ for the sustainable production of DHBZ.

The mixing contributes to an increase in the reaction rates of H_2_O_2_ decomposition, phenol hydroxylation, and DHBZ production. Under stirring, the oxygen bubbles produced from the HO*_x_*· species in autoscavenging radical reactions are moved out of the monolith channels faster in a fixed-bed reactor, and they do not hinder the transport of the reactants to the Fe active sites. Therefore, in the MSR, the kinetic model that describes the chemical reactions consists of power law equations, with first-order for each species. However, for the MMR and MFB, the best-fit model consists of hyperbolic equations where the denominator includes the resistance of the H_2_O_2_ transport to the active sites.

As the mixing degree increases, the efficiency in the H_2_O_2_ consumption and the tar production increases. The backmixing has an adverse effect on the phenol selectivity to DHBZ because a higher residence time favors the overoxidation of DHBZ to tar products.

The wall porosity of the 3D Fe/SiC structured catalyst and not the backmixing in the reactor is the main factor for the Fe leaching. The higher the wall porosity, the higher susceptibility to Fe leaching. Only in the case of the MMR (ε_wall_ (%) = 21 for mesh and 16 for monoliths), the reaction rates are affected by the catalytic effect of the Fe leaching.

Both the tar deposits on the catalyst surface and Fe leaching negatively affect the stability of the 3D Fe/SiC catalyst. The MSR is most affected by the tar and the MMR by Fe leaching. The MFB seems to be the most convenient reactor because, although it exhibits similar outstanding yields to DHBZ compared with the MSR and the MMR (*Y*_DHBZ_ = 29% at 80 °C in water), it provides the longest-standing performance.

## Supporting Information Available

Details of the different 3D Fe/SiC reactors studied; relationship between conversions and concentration of leached Fe in the liquid phase; parity plot and standard deviations of the apparent kinetic rate constant values estimated; and also, details for the calculation of L–S mass transfer coefficients and external mass transfer analysis (PDF)

## Symbols

*a*: distance between in-plane adjacent rods (mm)
*A*_ext_: external area (m^2^ g^–1^)
*a*_v_: channel interface area (mm^–1^)
*Ca*: Carberry number
*C*_BB_: bromophenol blue concentration (g L^–1^)
*C*_CAT_: catalyst concentration (g L^–1^)
*C*_i_: reactant concentration (mol L^–1^)
*C*_j_: product concentration (mol L^–1^)
*D*: diameter (mm)
*d*_H_: hydraulic diameter (mm)
*E*(*t*): residence time distribution function
*E*(θ): dimensionless residence time distribution function
*F*(*t*): cumulative distribution function
*H*: height (mm)
H_2_O_2EFF_: H_2_O_2_ efficiency (%)
*K*: kinetic parameter related to H_2_O_2_ adsorption
*k*_i_: apparent kinetic rate constant for reactants
*k*_j_: apparent kinetic rate constant for products
*k*_s_: L–S mass transfer coefficient (m s^–1^)
*L*: reactor length
*N*: number of moles
*Pe*: Péclet number
*Q*_L_: liquid flow (ml min^–1^)
*R*^2^: coefficient of determination
*Re*: Reynolds number
(−*r*_i_)_obs_: observed reaction rate for reactant i (mol L^–1^ s^–1^)
(*r*_j_)_obs_: observed reaction rate for product j (mol L^–1^ s^–1^)
*S*_BET_: specific surface area (m^2^ g^–1^)
*Sc*: Schmidt number
*Sh*: Sherwood number
*S*_j_: phenol selectivity to product j (%)
*T*: temperature (°C)
*t*_m_: mean residence time (min)
*V*_L_: liquid volume (ml)
*W*: weight (g)
*W*_CAT_: catalyst weight (g)
wt. loss: weight loss (%)
*X*_i_: reactant conversions (%)
*Y*_j_: phenol yield to product j (%)
%Fe_leached_: percentage of leached Fe
%TOC_adsorbed_: percentage of carbon adsorbed on the catalyst surface (%)

## Greek Symbols

δ_wall_: wall channel thickness (μm)
ε_total_: open total porosity (%)
ε_wall_: open rod porosity (%)
η: cell density (cell cm^–2^)
θ: dimensionless time
ρ_geo_: geometrical density (g cm^–3^)
ρ_bulk_: bulk density (g cm^–3^)
σ_t_^2^: variance (min^2^)
τ: space time (g_CAT_ h L^–1^)
χ^2^: chi-square

## Subscripts and Supercripts

0: initial conditions
b: conditions in the liquid phase
i: reactant
j: product
*t*: given reaction time conditions

## Abbreviations

BB: bromophenol blue
BQ: *p*-benzoquinone
CAD: computer-aided design
CTL: catechol
DHBZ: dihydroxybenzenes
DAD: diode array detection
DTA: differential thermal analysis
HQ: hydroquinone
MFB: monolithic fixed-bed
MMR: multimesh fixed-bed reactor
MSR: monolithic stirrer reactor
PLA: polylactic acid
POCs: periodic open-cellular structures
RSL: resorcinol
RSS: residual sum of squares
RTD: residence time distribution
S-MMR: separated multimesh fixed-bed reactor
SR: slurry reactor
TAR: unidentified product
TGA: thermogravimetric analysis
TOC: total organic carbon
