# Supplementary material for: Structured Reactors Based on 3D Fe/SiC Catalysts: Understanding the Effects of Mixing
Source: Ind Eng Chem Res. 2022 Aug 8;61(32):11678–90. doi: 10.1021/acs.iecr.2c01611 (PMC9828541; doi:10.1021/acs.iecr.2c01611)
Supplement: Supplementary file 1 — ie2c01611_si_001.pdf [file ie2c01611_si_001.pdf]

Supporting Information

Structured reactors based on 3D Fe/SiC catalysts: understanding the effects of mixing

Gonzalo Vega[1,*], Asuncion Quintanilla[1,*], Pablo López[1], Manuel Belmonte[2], Jose A. Casas[1]

[1] Department of Chemical Engineering, Universidad Autónoma de Madrid, Campus de Cantoblanco, C/Francisco Tomás y Valiente 7, 28049 Madrid, Spain

[2] Institute of Ceramics and Glass (ICV-CSIC), Campus de Cantoblanco, C/Kelsen 5, 28049 Madrid, Spain

*Email: gonzalo.vega@uam.es; asun.quintanilla@uam.es

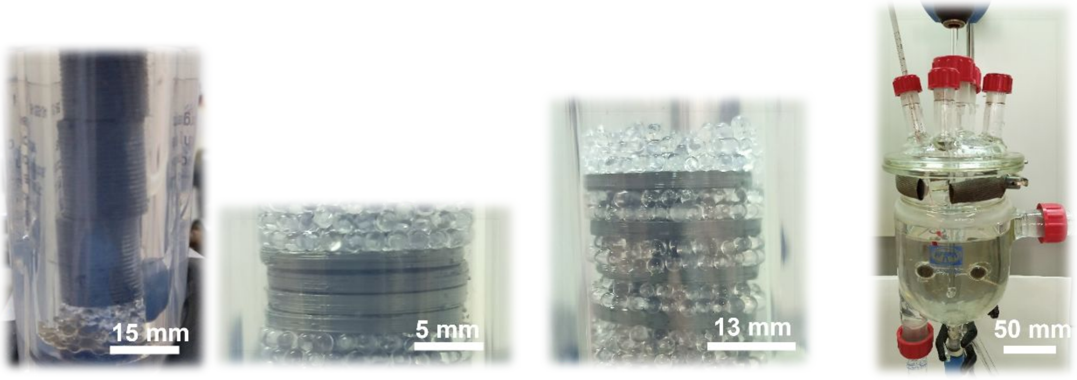

Figure S1. Photographs of the different 3D Fe/SiC reactors studied: MFB (a), MMR (b), S-MMR (c) and MSR (d).

Table S1. Main characteristics of the 3D Fe/SiC reactors used in this work

Type of reactor	No. structures	W_{CAT} (g)	V_L (mL)	C_{CAT}[a] (g L^{-1})
Monolithic fixed bed (MFB)	3	3.9	3.7	1054
Multi-mesh fixed bed (MMR)	4	2.4	2.5	960
Separated multi-mesh fixed bed (S-MMR)	4	2.4	6.5[b]	369
Monolithic stirrer (MSR)	2	2.6	725	4
Slurry (SR)	-	2.6	725	4

[a] calculated as W_{CAT} V_L^{-1} being the $V_L = \varepsilon_{TOTAL}$ V_R

[b] experimentally measured by filling the reactor with water

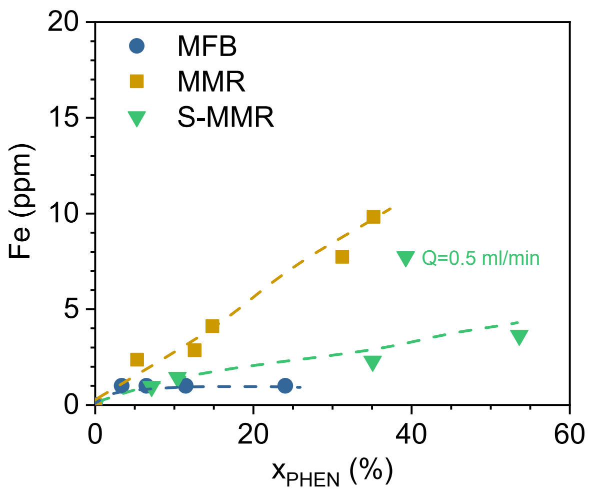

Figure S2. Relationship between H_2O_2 or phenol conversion and the concentration of leached Fe in the liquid phase in the fixed bed reactors.

Table S2. Characterization results for the used 3D Fe/SiC catalysts in the different reactors.

Reactor*	S_{BET} ($m^2\ g^{-1}$)	A_{EXT} ($m^2\ g^{-1}$)	wt. loss (%)**	C (%wt.)
MFB	34 (39)	13 (27)	0.52 (0.52)	1.4 (1.4)
MMR	22 (25)	16 (25)	0.48 (0.52)	1.9 (1.4)
S-MMR	28 (25)	22 (25)	0.71 (0.52)	1.9 (1.4)
MSR	21(39)	11 (27)	1.01(0.52)	3.2 (1.4)
SR	22 (39)	9 (27)	1.50 (0.52)	3.5 (1.4)

*Fresh values in brackets. **Weight loss measured by TGA

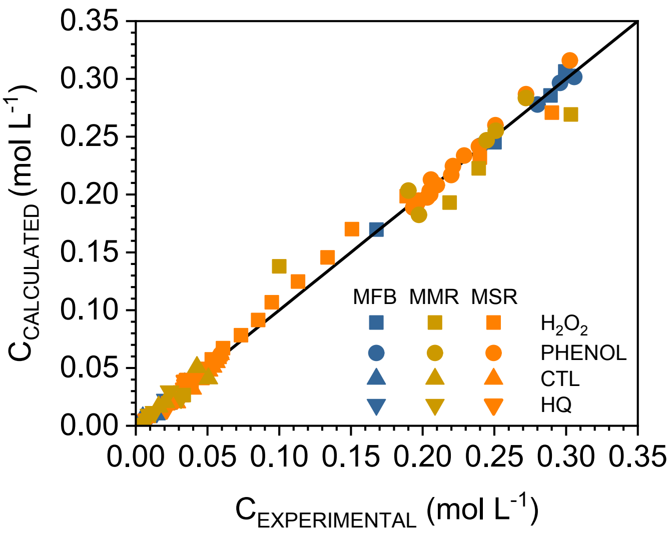

Figure S3. Parity plot of experimental and calculated concentrations by the kinetic models provided in Table 3.

Table S3. Apparent kinetic rate constant values with the standard deviations calculated for reactants and products in the phenol hydroxylation with H_2O_2 over 3D Fe/SiC catalysts in the different reactors

**Reactor**	**$(-r_{H2O2})$ (mol g_{cat}^{-1} h^{-1})**	**$(-r_{PHENOL})$ (mol g_{cat}^{-1} h^{-1})**
**MFB**	$\frac{(1.0\cdot10^{-2} \pm 3.0\cdot10^{-4})C_{H2O2}}{1 + 11.8\ C_{H2O2}}$	$\frac{(1.7\cdot10^{-2} \pm 6.4\cdot10^{-4})C_{H2O2}\ C_{PHENOL}}{1 + 11.8\ C_{H2O2}}$
**MMR**	$\frac{(3.9\cdot10^{-2} \pm 3.1\cdot10^{-3})C_{H2O2}}{1 + 11.84\ C_{H2O2}}$	$\frac{(6.3\cdot10^{-2} \pm 6.1\cdot10^{-3})C_{H2O2}\ C_{PHENOL}}{1 + 11.84\ C_{H2O2}}$
**MSR**	$(1.7\cdot10^{-1} \pm 3.3\cdot10^{-3})C_{H2O2}$	$(3.4\cdot10^{-1} \pm 6.9\cdot10^{-3})C_{H2O2}\ C_{PHENOL}$

**Reactor**	**r_{CTL} (mol g_{cat}^{-1} h^{-1})**	**r_{HQ+BQ} (mol g_{cat}^{-1} h^{-1})**
**MFB**	$\frac{(8.5\cdot10^{-3} \pm 7.5\cdot10^{-4})C_{H2O2}\ C_{PHENOL}}{1 + 11.8\ C_{H2O2}}$	$\frac{(5.3\cdot10^{-3} \pm 5.8\cdot10^{-4})C_{H2O2}\ C_{PHENOL}}{1 + 11.8\ C_{H2O2}}$
**MMR**	$\frac{(3.0\cdot10^{-2} \pm 2.7\cdot10^{-3})C_{H2O2}\ C_{PHENOL}}{1 + 11.84\ C_{H2O2}}$	$\frac{(1.8\cdot10^{-2} \pm 2.1\cdot10^{-3})C_{H2O2}\ C_{PHENOL}}{1 + 11.84\ C_{H2O2}}$
**MSR**	$(1.5\cdot10^{-1} \pm 3.2\cdot10^{-3})C_{H2O2}\ C_{PHENOL}$	$(9.8\cdot10^{-2} \pm 3.3\cdot10^{-3})C_{H2O2}\ C_{PHENOL}$

Calculations of L-S mass transfer coefficient (k_{MT}) for phenol and H_2O_2 in the different reactors at 80 ºC:

$$\mathrm{Sh} = 2.98\left[1 + 0.095\frac{\mathrm{d_H}}{\mathrm{L}}\mathrm{Re\,Sc}\right]^{0.45} \quad \text{for } \mathrm{L} \leq 0.05\ \mathrm{Re\,Sc\,d_H}$$

$$\mathrm{Sh} = 2.98 \text{ for } \mathrm{L} > 0.05\ \mathrm{Re\,Sc\,d_H}$$

$$\mathrm{Re} = \frac{\mathrm{u \cdot d_H \cdot \rho}}{\mu} \qquad \mathrm{Sc} = \frac{\mu}{\rho \cdot \mathrm{D_{m,i}}} \qquad Sh = \frac{\mathrm{k_{MT} \cdot d}_H}{\mathrm{D_{m,i}}}$$

Table S4a. Values of L-S mass transfer coefficient for phenol and H_2O_2 in monolithic reactors

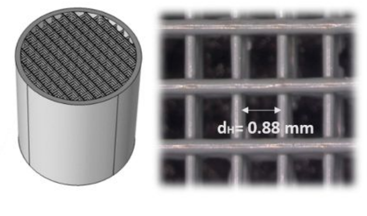

DIMENSIONS		
Hydraulic diameter	d_H (mm)	0.88
Reactor lenght	L (mm)	44.40
Monolith diameter	D (mm)	13.80
Monolith cross section	S (mm^2)	149.6
Monolith cross section	S (m^2)	1.50E-04
Cell density	η ($cells/cm^2$)	74
Number of cells	(cells/monolith)	68
Open cross section	Sc (mm^2)	0.387
Open cross section	Sc (m^2)	3.87E-07
Channel Interfacial area	a_v (mm^{-1})	26

REACTION MEDIA PROPERTIES AT 80 ºC	
WATER AS SOLVENT	
Pressure (atm)	1
M PHENOL (g/mol)	94
M_{H2O2} (g/mol)	34
T (ºC)	80
ρ_{water} (kg/m^3)	979.2
μ_{water} (kg/m·s)	4.34 E-04
$D_{m,\ FENOL}$ (m^2/s)	2.00E-09
$D_{m,\ H2O2}$ (m^2/s)	1.20E-06

Monolithic fixed bed reactor												
Q (ml/min)	Q (m^3/s)	u=Q/S (m/s)	Re	Sc_{PHENOL}	Sc_{H2O2}	$0.05ReScd_H$ (mm)	Sh_{PHENOL}	Sh_{H2O2}	$k_{MT,\ PHENOL}$ (mm/s)	$k_{MT,\ H2O2}$ (mm/s)	$k_{MT} \cdot a_{v,\ PHENOL}$ (s^{-1})	$k_{MT} \cdot a_{v,\ H2O2}$ (s^{-1})
0.25	4.17E-09	2.79E-05	0.055	221.61	369.35	0.90	2.98	2.98	0.0068	0.0041	**0.179**	**0.107**
0.5	8.33E-09	5.57E-05	0.111	221.61	369.35	1.80	2.98	2.98	0.0068	0.0041	**0.179**	**0.107**
1	1.67E-08	1.11E-04	0.221	221.61	369.35	3.60	2.98	2.98	0.0068	0.0041	**0.179**	**0.107**
2	3.33E-08	2.23E-04	0.442	221.61	369.35	7.19	2.98	2.98	0.0068	0.0041	**0.179**	**0.107**
						always lower than L						

Monolithic stirrer reactor										
u (m/s)	Re	Sc_{PHENOL}	Sc_{H2O2}	$0.05ReScd_H$ (mm)	Sh_{PHENOL}	Sh_{H2O2}	$k_{MT,\ PHENOL}$ (mm/s)	$k_{MT,\ H2O2}$ (mm/s)	$k_{MT} \cdot a_{v,\ PHENOL}$ (s^{-1})	$k_{MT} \cdot a_{v,\ H2O2}$ (s^{-1})
5.50E-02	109.201	221.61	369.35	1774.67	27.34	34.36	0.0621	0.0468	**1.64**	**1.24**

Table S4b. Values of L-S mass transfer coefficient for phenol and H_2O_2 in mesh reactors

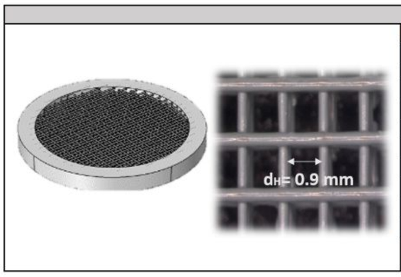

DIMENSIONS		
Hydraulic diameter	d_H (mm)	0.90
Reactor lenght	L (mm)	8.20
Monolith diameter	D (mm)	24.30
Monolith cross section	S (mm^2)	463.8
Monolith cross section	S (m^2)	4.64E-04
Cell density	η (cells/cm^2)	53
Number of cells	(cells/monolith)	225
Open cross section	Sc (mm^2)	0.405
Open cross section	Sc (m^2)	4.05E-07
Channel Interfacial area	a_v (mm^{-1})	21.4

REACTION MEDIA PROPERTIES AT 80 ºC	
WATER AS SOLVENT	
Pressure (atm)	1
M PHENOL (g/mol)	94
M_{H2O2} (g/mol)	34
T (ºC)	80
ρ_{water} (kg/m^3)	979.2
μ_{water} (kg/m·s)	4.34 E-04
$D_{m, FENOL}$ (m^2/s)	2.00E-09
$D_{m, H2O2}$ (m^2/s)	1.20E-06

Multimesh reactor												
Q_T (ml/min)	Q (m^3/s)	u0 (m/s)	Re	Sc_{PHENOL}	Sc_{H2O2}	$0.05ReScd_H$ (mm)	Sh_{PHENOL}	Sh_{H2O2}	$k_{MT, PHENOL}$ (mm/s)	$k_{MT, H2O2}$ (mm/s)	$k_{MT} \cdot a_{v, PHENOL}$ (s^{-1})	$k_{MT} \cdot a_{v, H2O2}$ (s^{-1})
0.25	4.17E-09	8.98E-06	0.018	221.61	369.35	0.30	2.98	2.98	0.0066	0.0040	**0.139**	**0.083**
0.5	8.33E-09	1.80E-05	0.036	221.61	369.35	0.61	2.98	2.98	0.0066	0.0040	**0.139**	**0.083**
1	1.67E-08	3.59E-05	0.073	221.61	369.35	1.21	2.98	2.98	0.0066	0.0040	**0.139**	**0.083**
2	3.33E-08	7.19E-05	0.146	221.61	369.35	2.43	2.98	2.98	0.0066	0.0040	**0.139**	**0.083**
						always lower than L						

Table S5. Initial apparent reaction rates, mass transfer rates and Ca numbers for phenol, H_2O_2, catechol and hydroquinone in the different reactors.

Species	Reactor	Initial reaction rates (mol g^{-1} h^{-1})	Initial mass transfer rates (mol L^{-1} s^{-1})	Ca
H_2O_2	MFB	$6.8\ 10^{-4}$	$3.2\ 10^{-2}$	$1.8\ 10^{-3}$
	MMR	$2.6\ 10^{-3}$	$2.5\ 10^{-2}$	$3.6\ 10^{-2}$
	MSR	$5.2\ 10^{-3}$	0.37	$8.0\ 10^{-6}$
Phenol	MFB	$3.4\ 10^{-4}$	$5.3\ 10^{-2}$	$6.1\ 10^{-3}$
	MMR	$1.3\ 10^{-3}$	$4.2\ 10^{-2}$	$1.0\ 10^{-2}$
	MSR	$3.0\ 10^{-3}$	0.5	$6.0\ 10^{-6}$
CTL*	MFB	$1.7\ 10^{-4}$	$5.3\ 10^{-2}$	$9.2\ 10^{-4}$
	MMR	$5.9\ 10^{-4}$	$4.2\ 10^{-2}$	$5.0\ 10^{-3}$
	MSR	$1.3\ 10^{-3}$	0.5	$2.7\ 10^{-6}$
HQ*	MFB	$1.1\ 10^{-4}$	$5.3\ 10^{-2}$	$5.7\ 10^{-4}$
	MMR	$3.5\ 10^{-4}$	$4.2\ 10^{-2}$	$2.9\ 10^{-3}$
	MSR	$9.1\ 10^{-4}$	0.5	$1.8\ 10^{-6}$

*It is assumed the same mass transfer coefficient values for phenol, CTL and HQ.
